# mGem: A tale as old as blood—do tick-borne pathogens exploit arthropod antioxidant defenses?

**DOI:** 10.1128/mbio.00650-26

**Published:** 2026-07-02

**Authors:** Kaylee A. Vosbigian, Chelsea A. Osbron, Brianna P. Steiert, Cameron G. Coyle, Ashley L. Warren, Dana K. Shaw

**Affiliations:** 1 Department of Veterinary Microbiology and Pathology, Washington State University 6760, Pullman, Washington, USA; The Ohio State University, Columbus, Ohio, USA

**Keywords:** tick-borne pathogens, antioxidants, oxidative stress, arthropod vectors, vector-borne diseases, host-pathogen interactions

## Abstract

Effective control of tick-borne disease begins by first understanding how ticks acquire, harbor, and transmit pathogens. Over their lifetime, ticks encounter many sources of oxidative stress-inducing stimuli. Here, we explore the factors contributing to oxidative stress in ticks—including blood digestion, pesticide exposure, and pathogen infection—and then discuss how ticks counter this stress by employing antioxidant defenses. We highlight how non-tick-borne pathogens manipulate the host antioxidant response for survival and speculate that tick-borne pathogens may be acting similarly in the arthropod. We conclude by conjecturing that the robust antioxidant defenses that ticks have evolved to withstand the stress associated with hematophagy may also be inadvertently supporting the pathogens they carry.

## PERSPECTIVE

Ticks are obligate blood feeders that remain a significant public health threat owing to their ability to transmit a diverse range of pathogenic microbes (1, 2). This blood-feeding lifestyle, termed hematophagy, is a distinct evolutionary adaptation that has evolved independently across arthropods on more than 20 different occasions (3). Blood is a rich source of proteins and lipids (4–7), but nutritional dependence on it comes with inherent physiological trade-offs. The process of blood meal digestion liberates heme and iron, which are toxic in large quantities and lead to the formation of reactive oxygen species (ROS) (8–13). In addition to blood feeding, arthropod vectors encounter environmental and physiological sources of oxidative stress, including xenobiotic exposure (14) and pathogen infection (15–21). To counter this stress, ticks have evolved robust antioxidant networks to minimize self-inflicted oxidative damage (19, 22–29).

**Fig 1:**
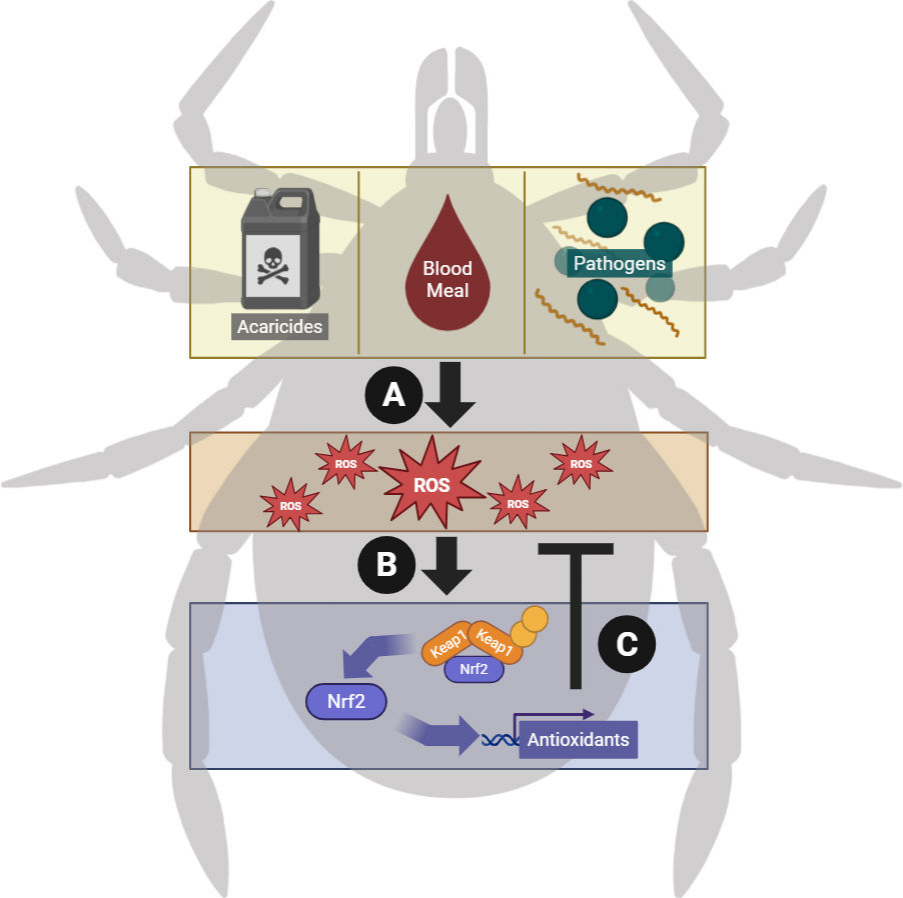
Schematic overview of oxidative stressors and responses in ticks. (**A**) External stimuli induce oxidative stress, (**B**) which activates Nrf2 control of the antioxidant response. (**C**) In turn, the antioxidant response decreases levels of ROS in cells.

Oxidative stress also exerts strong antimicrobial effects that are generally deleterious to pathogens (30). However, some microbes have evolved countermeasures to neutralize or suppress host-derived ROS (15, 31–51). Whether tick-borne microbes actively manipulate antioxidant defenses in the arthropod and whether this influences the vector’s competence to harbor and transmit pathogens is not known. Herein, we briefly outline oxidative stress and antioxidant responses. We then explore sources of oxidative stress encountered by ticks and cite examples of bacterial pathogens that manipulate host antioxidant defenses. Given the robust antioxidant network in ticks and the susceptibility of microbes to oxidative damage, we speculate that tick-borne pathogens may exploit antioxidant defenses to enable arthropod colonization. From this perspective, oxidative stress-mitigating mechanisms in the arthropod vector may act as a double-edged sword by protecting the tick but also inadvertently supporting pathogen persistence (Fig. 1).

## OXIDATIVE STRESS

ROS are highly reactive, oxygen-derived compounds capable of oxidizing a wide range of biological molecules. Many types of ROS exist, including free radicals such as superoxide anion (O_2_^−•^) and hydroxyl radical (HO^•^), as well as nonradicals like hydrogen peroxide (H_2_O_2_) and ozone (O_3_) (52). ROS are routinely produced endogenously through multiple mechanisms, with notable sources being the mitochondrial electron transport chain (ETC) (53), endoplasmic reticulum (ER) oxidoreductin-1 (ERO1) (54), and NADPH oxidases (NOX) (55). The role of ROS is most well characterized in phagocytic immune cells such as neutrophils (56) and macrophages (57), which rapidly generate large quantities of ROS. Although ticks do not have direct, functional equivalents to macrophages and neutrophils, they do have phagocytic immune cells, known as hemocytes, that play important roles in hematophagy and immune defense (58, 59). During phagocytosis, hemocytes also generate ROS, similar to mammalian phagocytes (60). However, ROS signaling plays a far broader biological role than the oxidative bursts characteristic of phagocytic cells.

ROS production increases in response to homeostasis-disrupting stimuli such as infection, ionizing radiation, heavy metals, pollutants, and chemotherapeutics (61–65). In these scenarios, ROS serve as regulatory signaling molecules in processes like metabolism, transcription, protein homeostasis, proliferation, and differentiation (66). During infection in mammals, ROS can also influence cytokine production, autophagy, apoptosis, extracellular trap formation, and immune cell activation (67, 68). While many of these immune processes are conserved in arthropods, it has not yet been empirically defined how ROS influences these pathways in ticks. However, the antimicrobial properties of ROS are most evident at high concentrations, which cause widespread damage to proteins, lipids, and nucleic acids—a phenomenon called oxidative stress.

Microbial invaders are not the sole targets of ROS-mediated damage. Oxidative stress can injure host tissues as well by causing genome instability, ER stress, and the production of damage-associated molecular patterns (DAMPs) (69). Damage to cellular membranes can occur when ROS attack polyunsaturated fatty acids. Oxygen radicals will remove electrons from lipids, producing lipid peroxyl radicals that then attack neighboring fatty acids, thereby creating a chain reaction (70). Consequently, accumulation of ROS can lead to cellular dysfunction, tissue damage, and chronic inflammation (69, 71). It is therefore critical to balance the antimicrobial properties of ROS with strategies that minimize collateral damage to host tissues. For this reason, cellular antioxidant responses are essential for regulating ROS levels and preserving reduction-oxidation (redox) homeostasis (72–74).

## ANTIOXIDANT DEFENSES

Cellular antioxidant responses are carried out by a diverse suite of enzymes that work synergistically to protect the cell from ROS. These include superoxide dismutases (SODs) (75), catalases (CATs) (76), glutathione S-transferases (GSTs) (77), and several selenoproteins such as thioredoxin (Trx) reductases (78), glutathione (GSH) peroxidases (GPxs) (79), and peroxiredoxins (Prxs) (80). SODs catalyze the conversion of two superoxide anions (O_2_^−•^) and two protons to form hydrogen peroxide (H_2_O_2_) and oxygen (O_2_) (75), thereby converting the highly reactive superoxide radical into its nonradical counterpart. Hydrogen peroxide is subsequently reduced to water by CATs (76), GPxs (79), and Prxs (80). Of these, GPxs and Prxs require a reducing substrate, with GPxs typically using GSH and Prxs using Trx. Notably, both GPxs and Prxs can reduce other substrates, including lipid hydroperoxides and alkyl hydroperoxides, respectively (79, 80). Trx reductases use NADPH to regenerate reduced Trx, which then directly reacts with oxidized proteins (81). GSTs function predominantly as detoxifying enzymes by conjugating GSH to xenobiotics, which facilitates their expulsion from the cell; however, some GSTs also have GPx activity that reduces lipid hydroperoxides (77, 82).

Antioxidant gene expression is regulated by transcription factors, including Nrf2 (Nuclear factor erythroid 2-related factor 2). Nrf2 is a basic leucine zipper (bZIP) transcription factor that is well conserved across species and is considered the master regulator of antioxidant responses (83). In homeostasis, Nrf2 is negatively regulated by Kelch ECH-associated protein 1 (Keap1) (Fig. 1). Keap1 binds to Nrf2 and recruits the ubiquitin ligases Cullin 3 (CUL3) and RING-box protein 1 (Rbx1), which constitutively ubiquitinate Nrf2, targeting it for proteasomal degradation (84–86). Nrf2 can be activated through several different processes. In the presence of ROS, oxidation of redox-sensitive cysteine residues on Keap1 causes dissociation from Nrf2 (84, 86). Alternatively, the stress-induced protein p62 can competitively bind to Keap1, thereby interrupting interactions with Nrf2 (87). Direct phosphorylation of Nrf2 by the unfolded protein response receptor PKR-like ER kinase (PERK) can also occur, which causes Keap1 to disassociate (88). Once Nrf2 dissociates from Keap1, it can translocate to the nucleus and bind to antioxidant response elements (AREs) within targeted gene promoters (Fig. 1). This regulates transcriptional programs governing redox homeostasis and diverse cellular processes such as metabolism, autophagy, inflammation, mitochondrial biogenesis, and immune signaling (85, 89).

## THE STRESSFUL LIFESTYLE OF TICKS

Ticks have evolved as obligate blood feeders, which expose them to a range of physiological and environmental pressures that are unique to their biology. Blood is a rich source of nutrients that support metabolism and development in hematophagous arthropods (6, 90, 91), but blood-feeding comes with a distinct set of stressors (92). During feeding, the arthropod must cope with thermal stress due to a marked rise in internal temperature of approximately 15–20°C. Blood feeding also brings a massive influx of water and ions that cause osmotic stress (7, 90, 93, 94). Internally, blood digestion results in the accumulation of heme and iron, which can contribute to oxidative stress in tick tissues (6, 95, 96). Consistent with this, lipid peroxidation has been reported to increase in *Haemaphysalis longicornis* during blood feeding (27). To mitigate heme-associated stress, midgut cells contain specialized organelles known as hemosomes, which sequester and process free heme (95, 96). Externally, host inflammatory responses at the bite site, including oxidative processes (97–99), create a hostile feeding environment that ticks actively counteract through the secretion of salivary molecules that suppress inflammation, coagulation, wound healing, and vasoconstriction (22, 25, 100–102).

Beyond blood feeding, acaricides are another source of oxidizing damage that ticks encounter (14). Acaricide treatments are employed as a strategy to prevent tick attachment to mammalian hosts. Common acaricide classes include synthetic pyrethroids, amitraz, fipronil, organophosphates, and macrocyclic lactones (103). These chemicals typically target the nervous system to cause paralysis, though newer classes may hinder molting/development or cellular respiration (27). Pesticides also cause oxidative stress by altering cellular respiration and the homeostatic redox balance, resulting in the generation of ROS (14).

Throughout their lifecycle, ticks must also contend with oxidative stress resulting from microbial infection. Tick-borne pathogens can colonize ticks with incoming blood meals from infected hosts. The act of blood feeding itself provokes an increase in tick hemocyte numbers and subtypes, likely resulting from prohemocyte differentiation and/or granulocyte proliferation (58, 59, 104). Infection triggers the arthropod’s immune defenses and initiates bursts of ROS (19, 20, 105–107). In *Ixodes scapularis*, both *Borrelia burgdorferi* and *Anaplasma phagocytophilum* elicit this oxidative response (19, 20, 108). *Babesia bovis* provokes phagocytosis and production of ROS from *Rhipicephalus microplus* hemocytes (60). In response, genes associated with oxidative stress are upregulated. When *Anaplasma marginale*, *B. bovis*, *B. burgdorferi*, and *Rickettsia montanensis* infect ticks, *GST* expression is induced (109–113). *GST* is also upregulated in an *Amblyomma americanum* hemocyte subset when infected with *E. chaffeensis* (59). *Peroxinectin,* a cell adhesin and immune component associated with oxidative stress, is upregulated during TBEV infection in *Ixodes* cells (114). Despite immune restrictions, these pathogens persist throughout the tick’s lifecycle, suggesting that they can subvert or manipulate these arthropod defenses.

## PATHOGEN EXPLOITATION OF THE ANTIOXIDANT RESPONSE

Many pathogens have evolved mechanisms to hijack antioxidant responses for their benefit. Some bacterial pathogens specifically target the Nrf2 pathway including *Staphylococcus aureus*, *Streptococcus pneumoniae*, *Mycobacterium tuberculosis*, *Listeria monocytogenes*, *Escherichia coli*, *Helicobacter pylori*, *Legionella pneumophila*, *Pseudomonas aeruginosa*, *Salmonella typhimurium*, *Chlamydia trachomatis*, and *Coxiella burnetii* (40, 83, 115). However, the molecular mechanisms underlying bacterial manipulation of the Nrf2 pathway are still not well understood.

Neutralizing oxidative stress is especially critical for intracellular pathogens, which are entirely reliant on the host cell for survival. This requires carefully orchestrated manipulation of the host cell (116). One way that intracellular bacteria accomplish this feat is through the secretion of virulence factors known as effector proteins (117). Effector proteins are delivered through specialized secretion apparatuses to the host cell cytosol and function either individually or in concert with other effectors. Often, they mimic host proteins to manipulate signaling pathways. Effectors can also exhibit enzymatic activity that modifies host proteins, resulting in the disruption of signaling pathways. Certain effectors exhibit multifunctionality or participate in redundant pathways, adding complexity to their regulatory roles (116, 117).

Several obligate intracellular pathogens, including *C. trachomatis*, *C. burnetii,* and *L. pneumophila,* use secreted proteins to target the Nrf2 pathway (118–121). *C. trachomatis* induces host ROS within the first few hours of infection, but it is quickly shut down by the start of the bacterial developmental cycle (40). Infection causes upregulation of the antioxidant enzymes NAD(P)H quinone oxidoreductase 1 (NQO1) and heme oxygenase-1 (HO-1), both of which are Nrf2 regulated in mammals (118, 119). Chlamydial protein, Pgp3 (122), induces the nuclear translocation of Nrf2 and subsequent upregulation of NQO1 and HO-1 (118, 119). Stimulating infected host cells with Pgp3 also potentiates an antioxidant response and enhances *C. trachomatis* growth by reducing oxidative stress and decreasing apoptosis (118, 119). Despite these observations, the molecular mechanism by which Pgp3 manipulates the Nrf2 pathway has not yet been elucidated.

*C. burnetii* also resists oxidative stress, enabling the establishment of its replicative niche within the host (115). During *C. burnetii* infection, the Nrf2/Keap1 pathway is activated with sustained maintenance of Nrf2 levels and nuclear translocation (120). Winchell *et al*. observed that phosphorylated p62 accumulates in *C. burnetii*-infected cells, which binds Keap1 and frees Nrf2 (120). p62 accumulation localized around the *C. burnetii-*containing vacuole, which was dependent on the type IV secretion system (T4SS), suggesting that this is a bacterial-directed process (123). Which effector or collection of effectors is involved in manipulating this process has not been defined. However, one *C. burnetii* effector has been identified to manipulate the antioxidant response. Mitochondrial *Coxiella* effector protein F (MceF) recruits glutathione peroxidase 4 (GPX4) to the mitochondria during *C. burnetii* infection (124), which reduces mitochondrial ROS production and protects the host from ROS-induced cell death. Notably, GPX4 is Nrf2-regulated (125) and may be a future area of inquiry.

The secreted effectors associated with *L. pneumophila’s* modulation of the antioxidant response are more defined. Nrf2 is differentially targeted by *L. pneumophila* depending on the stage of infection, which is orchestrated by effector proteins. The bacterium inhibits Nrf2 early in infection with SidE enzymes (SdeA, SdeB, SdeC, and SidE) that catalyze phosphoribosyl-linked (PR) ubiquitination of p62 (121, 126, 127). This blocks the interaction between p62 and Keap1, resulting in ubiquitination and degradation of Nrf2 and a delayed antioxidant response (121). After infection is established, secreted effectors DupA and DupB act as deubiquitinases for PR ubiquitination (128). The reversal in PR ubiquitination of p62 allows for p62 to bind Keap1, thereby releasing Nrf2 and allowing it to translocate to the nucleus, where it upregulates antioxidant gene expression (121).

**Fig 2:**
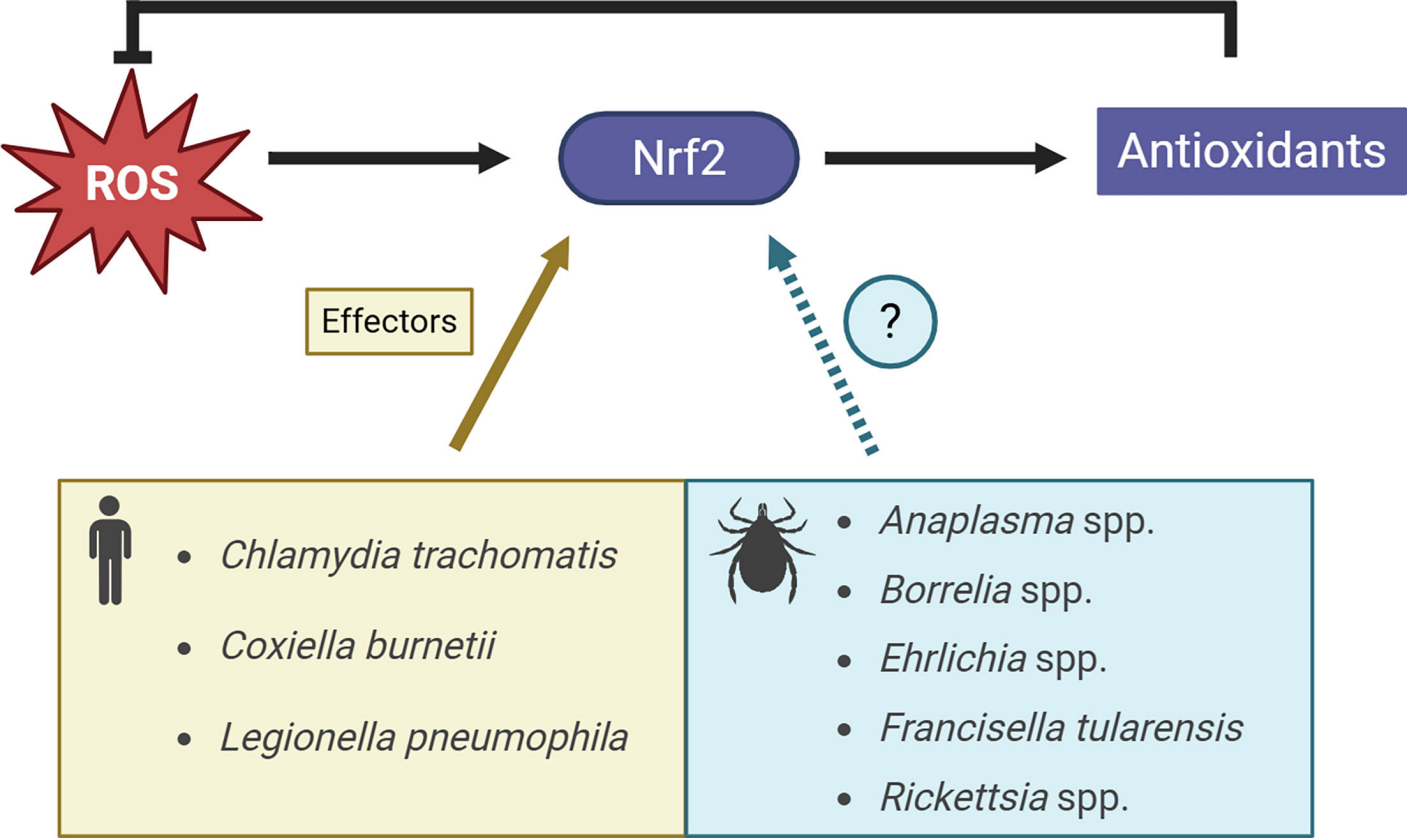
Pathogens and the antioxidant response. ROS activates Nrf2, which upregulates the expression of antioxidant genes to restore redox homeostasis. The yellow box highlights non-tick-borne pathogens that modulate Nrf2 signaling using secreted effector proteins. The blue box contains tick-borne pathogens that are known or suspected to benefit from Nrf2 activity, potentially through direct modulation of the pathway.

How tick-borne pathogens survive oxidative stress within the arthropod vector is poorly understood at the mechanistic level. However, tick-borne bacterial pathogens, including *Anaplasma* spp., *Rickettsia* spp., *Ehrlichia* spp., and *Francisella tularensis*, also encode secretion systems and a plethora of secreted effector proteins (129). Moreover, several of these microbes suppress ROS in mammalian hosts (15, 17, 18, 130–132). *Ehrlichia chaffeensis* uses EtpE (entry-triggering protein of *Ehrlichia*) to not only induce bacterial entry into mammalian cells but to also block ROS production (133). *A. phagocytophilum* also suppresses oxidative bursts in the mammalian host (33–37, 51). Despite growing insight into how these pathogens impact mammalian hosts, how they evade oxidative stress in arthropods is comparatively less understood. One previous report demonstrated that *A. phagocytophilum* activates the l-tryptophan pathway in tick cells, subsequently upregulating metabolites that reduce the buildup of ROS (130), yet the mechanism by which the bacterium initiates this pathway remains undefined. Given that many tick-borne pathogens suppress ROS in mammals, it is possible, if not probable, that these microbes also directly manipulate the arthropod antioxidant response (129). Whether this is through direct manipulation of the tick Nrf2 pathway remains unknown (Fig. 2).

## TICK-BORNE PATHOGENS AND THE VECTOR ANTIOXIDANT RESPONSE

For most microbes, oxidative stress is a hostile and potentially lethal challenge. In ticks, depleting the antioxidant thioredoxin reductase reduces the natural microbiome (134, 135). Similarly, harmful effects are seen on tick-borne pathogens. For instance, knocking down the expression of *catalase* or *selenoprotein P* in adult *Amblyomma maculatum* decreased transovarial transmission of *Rickettsia parkeri* (24, 25). Additionally, knocking down *selenoprotein P* or *Sec insertion sequence binding protein 2* (*SBP2*) in these ticks also impacted the expression of other antioxidant genes (24). When tick cell cultivation media is supplemented with the antioxidant N-acetyl cysteine, *A. phagocytophilum* levels increase in *Ixodes* tick cells (19, 108). During tick feeding, *B. burgdorferi* benefits from Salp25, an *Ixodes* saliva protein with homology to Prxs (136). Additionally, Selenoprotein K plays an essential role in *B. burgdorferi* tick colonization (137) by regulating oxidative and ER stress during prolonged feeding in ticks (134). Decreasing the expression of *GST* through RNA interference reduced *A. marginale* colonization in *R. microplus* cells (138) and in *Dermacentor variabilis* ticks (113, 139). These studies collectively demonstrate that tick-borne pathogens capitalize on arthropod antioxidant defenses to support infection and persistence.

Given that infection changes the arthropod redox environment, it is reasonable to speculate that Nrf2 may be actively targeted or manipulated by tick-borne pathogens. One study found that both *A. phagocytophilum* and *B. burgdorferi* activate the *I. scapularis* PERK/eIF2α/Nrf2 signaling axis, which enhances their survival (19). Silencing genes in this pathway led to decreased pathogen acquisition by *I. scapularis* larvae and diminished pathogen survival in molted nymphs. This survival defect was also observed in tick cell culture and could be rescued with the addition of exogenous antioxidants, demonstrating that the PERK/eIF2α/Nrf2 pathway supports *A. phagocytophilum* and *B. burgdorferi* by detoxifying ROS (19). Whether this is a pathogen-directed process or a cytoprotective response initiated by the host remains undetermined.

## CONCLUDING REMARKS

Tick-borne pathogens encounter molecular stressors in the arthropod that differ substantially from those in mammalian hosts. This requires specialized adaptations to enable persistence in the arthropod. How these microbes overcome such challenges to colonize the tick and ensure transmission to a new host is not well understood. Recent work has highlighted how cellular stress responses interact with arthropod immune pathways and shape vector competence (19, 20, 140–142). With this in mind, understanding whether tick-borne pathogens induce or manipulate these pathways to exploit the antioxidant response could provide insights for developing new strategies to curb tick-borne disease.
